# Impact of Crystal Habit on the Dissolution Rate and In Vivo Pharmacokinetics of Sorafenib Tosylate

**DOI:** 10.3390/molecules26113469

**Published:** 2021-06-07

**Authors:** Chi Uyen Phan, Jie Shen, Kaxi Yu, Jianming Mao, Guping Tang

**Affiliations:** 1 Faculty of Chemical Technology—Environment, The University of Danang—University of Technology and Education, Danang 550000, Vietnam; 2 Department of Chemistry, Zhejiang University, Hangzhou 310028, China; shenjie1003@zju.edu.cn (J.S.); yukaxi@zju.edu.cn (K.Y.); 3150102050@zju.edu.cn (J.M.)

**Keywords:** crystal habit, sorafenib tosylate, dissolution rate, pharmacokinetics

## Abstract

The dissolution rate is the rate-limiting step for Biopharmaceutics Classification System (BCS) class II drugs to enhance their in vivo pharmacokinetic behaviors. There are some factors affecting the dissolution rate, such as polymorphism, particle size, and crystal habit. In this study, to improve the dissolution rate and enhance the in vivo pharmacokinetics of sorafenib tosylate (Sor-Tos), a BCS class II drug, two crystal habits of Sor-Tos were prepared. A plate-shaped crystal habit (ST-A) and a needle-shaped crystal habit (ST-B) were harvested by recrystallization from acetone (ACN) and n-butanol (BuOH), respectively. The surface chemistry of the two crystal habits was determined by powder X-ray diffraction (PXRD) data, molecular modeling, and face indexation analysis, and confirmed by X-ray photoelectron spectroscopy (XPS) data. The results showed that ST-B had a larger hydrophilic surface than ST-A, and subsequently a higher dissolution rate and a substantial enhancement of the in vivo pharmacokinetic performance of ST-B.

## 1. Introduction

The Biopharmaceutics Classification System (BCS) categorizes pharmaceutical drugs into four classes based on their aqueous solubility and permeability behavior, including class I (high solubility, high permeability), class II (low solubility, high permeability), class III (high solubility, low permeability), and class IV (low solubility, low permeability) [1,2]. BCS class II and class IV drugs have a poor aqueous solubility and thus a low bioavailability. Improving the solubility of BCS class II and class IV drugs is therefore considered one of the toughest challenges in bioavailability enhancement [3,4].

**Figure 1:**
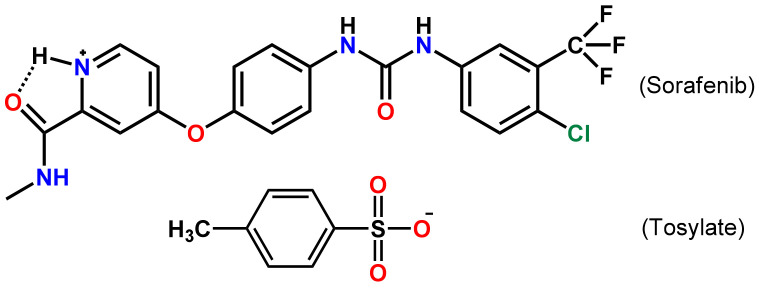
Molecular structure of sorafenib tosylate.

Sorafenib (Sor), an oral administration drug, has been used as a targeting therapeutic agent for a large range of tumor types via a dual mechanism of action. It is clinically approved by the US Food and Drug Administration (FDA) as a first-line treatment for patients with advanced renal cancer carcinoma, advanced hepatocellular carcinoma, etc. However, it is a BCS class II drug with a very poor aqueous solubility [5]. To improve its efficiency, sorafenib tosylate (Sor-Tos, Figure 1) has been used as the only clinical form instead of the free drug [6]. Compared to Sor, Sor-Tos has a higher solubility, a higher bioavailability, and a lower side effect [7,8], but its aqueous solubility is still low, leading to its slight absorption in the gastrointestinal tract [9,10]. Therefore, the dissolution rate plays a vital role in increasing the bioavailability of Sor-Tos, especially when a slight increase in the dissolution rate can produce a significant improvement in bioavailability [11].

The dissolution rate of drugs is affected by some common crystal characteristics such as polymorphism, particle size, and crystal habit [12,13]. Many studies have demonstrated the effect of polymorphism and particle size on dissolution rate [14,15]. Besides this, crystal habit, which is described as the external appearance of a crystalline solid, is also attracting attention in the pharmaceutical industry [16,17]. Crystal habit impacts the physicochemical properties and behaviors of a drug due to the anisotropic surface chemistry of different facets in crystalline material [18,19]. For example, the higher dissolution rate and oral bioavailability of celecoxib crystals in plate-shaped crystals than that in acicular crystals was attributed to their different hydrophilic surfaces [20]. The plate-shaped crystals of ticagrelor showed a higher hydrophilic nature than the needle-like crystals, resulting in a faster dissolution rate [21]. Moreover, crystal habit also influences industrial crystallization processes, such as filtration, washing, drying, packaging, etc. [12]. Factors that influence crystal habit include solvent, temperature, stirring rate of crystallization, degree of supersaturation, additives, rate of change of temperature, etc. The effect of solvent on the modification of the crystal habit is the most studied topic of focus [22].

In this study, we explored the effect of the crystal habit of Sor-Tos on its dissolution rate and in vivo pharmacokinetic behaviors. Two crystal habits of Sor-Tos were prepared by recrystallization from acetone and n-butanol. Their surface anisotropies were analyzed to evaluate the hydrophilic property using powder X-ray diffraction (PXRD), the MOLCAD program, face indexation, and X-ray photoelectron spectroscopy (XPS). Their dissolution rates and in vivo pharmacokinetic data were determined.

## 2. Results and Discussion

### 2.1. Morphology of Crystals

The solute in a supersaturated solution with solvents that have different polarity and viscosity can induce the formation of crystals with different morphology [22]. In different recrystallization conditions, the growth rate of crystal faces will be modified, resulting in the disappearance or appearance of some faces or variations in face dimensions, and thus the altered morphology of crystals [23].

**Figure 2:**
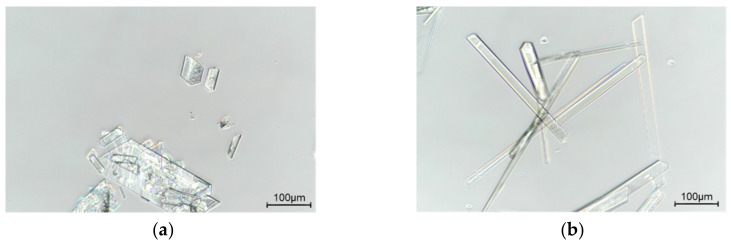
Compound micrographs of the crystal habit of Sor-Tos crystals grown from (**a**) acetone and (**b**) n-butanol.

In this research, to study the crystal habit of Sor-Tos, we recrystallized Sor-Tos from a supersaturated solution of a drug in two solvents with different polarity and viscosity, acetone, and n-butanol [24,25]. The obtained crystals (named ST-A and ST-B) were imaged (Figure 2). ST-A crystals presented a plate shape with an aspect ratio of 1:2~1:3, while ST-B crystals exhibited a needle shape with an aspect ratio of 1:10~1:20.

### 2.2. Differential Scanning Calorimetry (DSC)

DSC thermographs of ST-B and ST-A are depicted in Figure S1, which indicates that the two crystal habits remained similar. Their internal structures were not changed to other polymorphisms (different conformations) or solvate compounds.

### 2.3. Powder X-ray Diffraction (PXRD)

**Figure 3:**
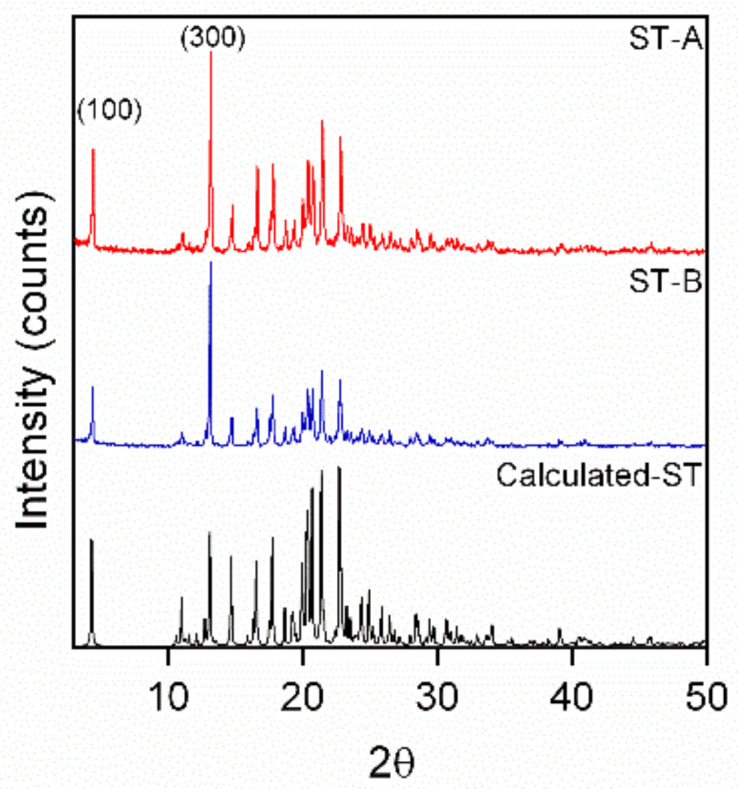
Overlay of PXRD patterns of ST-A, ST-B, and calculated ST.

The two crystal habits ST-A and ST-B used in the PXRD experiments were sieved through a 75 μm sieve. The PXRD patterns exhibited diffraction peaks at 2θ values of 4.4, 11.0, 13.2, 14.7, 16.6, 17.8, 20.4, 20.8, 21.5, and 22.9° (Figure 3), which were identical to the calculated patterns of Sor-Tos, indicating that the structure of the two Sor-Tos crystal habits was not changed (such as polymorphic change or solvate [26]).

The preferred crystal growth directions that impact the crystal sizes and habits could be determined by some higher peaks [21]. In these crystals, their relative peak intensities were varied (Table S1). The two highest diffraction peaks were 4.4^o^ (d = 19.99 Å) and 13.2° (d = 6.69 Å), corresponding to the (100) and its relevant (300) crystal planes, respectively. This result indicated that the dominant growth directions of Sor-Tos crystals were the (010) and (001) directions. In the two samples, the strongest intensity belonged to the diffraction peak of the (300) crystal plane. In other words, two crystal habits exposed the (100) crystal plane.

### 2.4. Face Indexation

Before determining face indexation of the two crystals, their space group and unit cell parameters were confirmed and found to agree with the cell parameters reported by Ravikumar et al. (P2_1_/c, *a* = 21.276Å, *b* = 9.116Å, *c* = 16.077Å, α = 90°, β = 108.143°, γ = 90°) [27]. The dominant face of each crystal was very different due to the varied growth rate. The crystal structure of Sor-Tos exhibited the hydrogen bond along the *b* axis [28]. Besides that, the π∙∙∙π planar stacking between two pyridinium rings (symmetry code was *(2-x, 2-y, 2-z)*, and the distance between the two planes was 3.921 Å) governed the crystal growth along the *c* axis. Thus, the crystals should grow fast along the (010) and/or (001) direction. The obviously faster growth along the (010) direction than the (001) or (100) direction led to a needle-shaped ST-B crystal.

**Figure 4:**
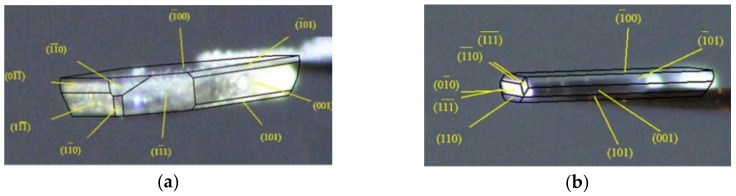
Face indexation data of (**a**) ST-A and (**b**) ST-B.

The faster growth along both (010) and (001) directions than the (100) direction led to a plate-shaped ST-A crystal. Therefore, (001) was the different dominant facet of the two crystal habits; the most dominant facet of ST-A was (100), while the dominant facets of ST-B were not only (100) but also (001) (Figure 4). In addition, from the face indexation data, % contributions of each facet to the total crystal surface (the relative abundance) could be determined. The total relative abundance of both (100) and (−100) faces of the two crystal habits were calculated to be about 69.8% and 58.1% for ST-A and ST-B, respectively. On the other hand, (001) and (00−1) contributed less in ST-A (14.9%) than ST-B (17.5%).

### 2.5. Molecular Modeling

**Figure 5:**
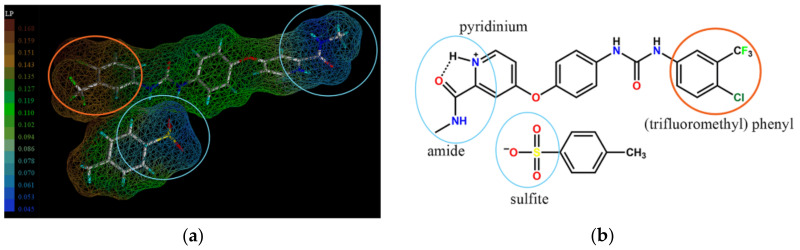
(**a**) LP analysis of Sor-Tos, moving from orange to blue on the scale, indicates increasing hydrophilic potential. (**b**) The corresponding molecular structure of Sor-Tos. The hydrophilic and hydrophobic moieties of Sor-Tos are shown in light blue and orange circles, respectively.

Figure 5 shows the hydrophilic and hydrophobic behavior of two parts of Sor-Tos by simulating LP on the surface of the molecule. The hydrophilic moieties are presented in blue shade (Figure 5a), and their corresponding functional groups are denoted in light blue circles (Figure 5b). By contrast, the hydrophobic moiety is displayed in orange shade (Figure 5a), and the corresponding functional group is presented in orange circle (Figure 5b). On the surface of the [Sor∙H]^+^ cation, the amide group and pyridinium ring were unambiguously responsible for polarity, while the (trifluoromethyl) phenyl ring functioned as a hydrophobic region. With regard to the tosylate anion, the hydrophilic and hydrophobic regions on its surface were attributed to the sulfite group and the methyl phenyl ring, respectively.

**Figure 6:**
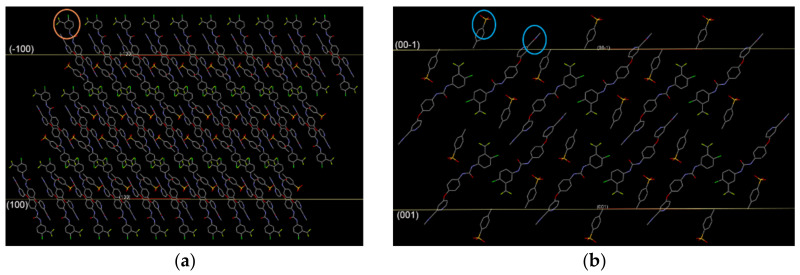
Molecular surface packing visualized along with the surface chemistry of (**a**) the (100) and (-100) as well as (**b**) (001) and (00-1) facets.

Moreover, visualization of the molecular surface packing of the Sor-Tos structure along the surface chemistry of dominant planes helped to define the most exposed chemical groups on the surface of these crystal facets (Figure 6). As mentioned above, the major planes contributing to the crystal surface were the (100) and (-100) planes. The inversely oriented Sor molecules on the (100) and (-100) facets displayed the hydrophobic chemical group (trifluoromethyl) phenyl ring, while they hindered the amide group (Figure 6a), resulting in a more hydrophobic surface. Yet, Sor molecules on the (001) and (00-1) facets exposed the sulfite and amide moieties, making the surface more hydrophilic. Taken together, ST-B crystals should be more hydrophilic than ST-A crystals.

### 2.6. X-ray Photoelectron Spectroscopy (XPS)

For a crystal, due to the structural anisotropy, the orientation of molecules is different on each facet [23]. Moreover, the different surface properties of the powders also originate from the discrepancy in the relative abundance of different facets, which is varied from crystal habit to crystal habit [8]. These differences in their surface chemistry can be determined by XPS.

Figure S2 indicates that the exposed chemical elements on the surface were fluorine (F), oxygen (O), nitrogen (N), carbon (C), chlorine (Cl), and sulfur (S). In addition, the qualities of the two powders were no different based on the similar peak shape and chemical shift. However, the two powders presented different elemental composition on the surface (Table 1). The surface of ST-B powder displayed a higher concentration of hydrophilic elements (O, N, S) and a lower concentration of hydrophobic elements (C, F, Cl) than that of ST-A. The ratios (O + N + S)/(C + F + Cl) accounting for surface polarity were 50.0 % and 51.7 % for ST-A and ST-B, respectively. This result showed that the surface of ST-B was more polar than that of ST-A, in accordance with the data analyzed from face index, relative abundance, PXRD, molecular modeling, and crystal structure visualization.

### 2.7. Dissolution Rate of Sor-Tos Crystal Habit in Water and Gastric Juice pH 1.2 Acid Solution

**Figure 7:**
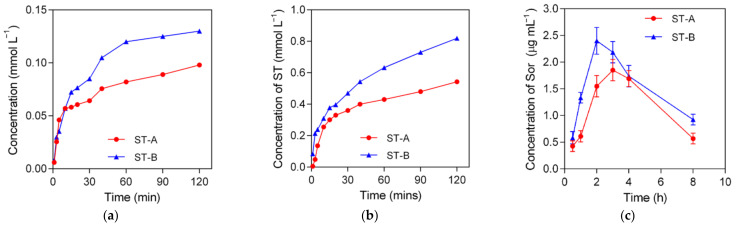
Dissolution rates of ST-A and ST-B in (**a**) water, (**b**) gastric juice pH 1.2 acid solution, and (**c**) in vivo pharmacokinetic profiles of ST-A and ST-B.

The dissolution rate of ST-A and ST-B, which have been passed through a 75 μm sieve, were measured at 1, 3, 5, 10, 15, 20, 30, 40, 60, 90, and 120 min in water and in gastric juice pH 1.2 acid solution (Figure 7a,b). The trends of the dissolution rate of ST-B and ST-A were similar in both media. In detail, ST-B exhibited significantly higher dissolution rates than ST-A. The solids dissolved rapidly within the first 30 min, which was followed by gradient dissolving. The solution did not reach equilibrium even after 2 h.

### 2.8. In Vivo Pharmacokinetics

To assess the in vivo pharmacokinetics of ST-A and ST-B, we obtained HPLC measurements of the drug concentration in plasma (Figure S3). The pharmacokinetic curves of ST-A and ST-B at a dosage of 27.5 mg kg^−1^ by oral administration are illustrated in Figure 7c. The results showed that the AUC (area under the curve) of Sor in ST-B significantly increased compared with that of ST-A. Furthermore, ST-B achieved a larger *C*_max_ at a shorter *T*_max_, while ST-A attained a smaller *C*_max_ at a longer *T*_max_ (Table 2). It could be concluded that ST-B manifested an enhanced pharmacokinetic performance with an increased concentration of Sor in the bloodstream, which might be attributed to the improved dissolution rate of ST-B.

## 3. Materials and Methods

### 3.1. Materials and Chemicals

Sorafenib tosylate (purity > 98%) was a kind gift from Eastchina Pharm. Co., Ltd., Zhejiang, China. It was initially characterized by PXRD. Acetone (ACN, MW = 58.08) and n-butanol (BuOH, MW = 74.12) were supplied by Sigma-Aldrich Co., Ltd., Santa Clara, CA, USA. All other materials and solvents were of analytical grade.

### 3.2. Crystallization Experiment

Different Sor-Tos crystal habits, including ST-A and ST-B, were prepared by recrystallization from acetone and n-butanol, respectively. The mixtures were heated to 60 °C with stirring until the solids dissolved completely, then the solutions were cooled to room temperature and the crystal habits of Sor-Tos were formed. The crystals were then filtered and dried at 50 °C for 2 h. The dried crystals were ground and sieved through a 75 μm sieve.

### 3.3. Characterization of Crystallized Solid Forms

#### 3.3.1. Optical Microscopy

Sor-Tos crystals were imaged for their crystal habits using Nikon DS-Ri2 (Nikon Corporation, Tokyo, Japan) at a magnification of 40×. A pre-calibrated stage micrometer was used to determine the aspect ratio of the Sor-Tos crystal habit.

#### 3.3.2. Differential Scanning Calorimetry (DSC)

A differential scanning calorimeter (TA DSC Q100) was used to perform thermal analysis of the samples of the two crystal habits. Each powder sample of approximately 3.0 mg was placed in an aluminum pan and heated at a rate of 10 °C min^−1^ under a nitrogen flow of 50 mL min^−1^ up to 300 °C.

#### 3.3.3. Powder X-ray Diffraction (PXRD)

A Rigaku D/Max-2550PC diffractometer was used to acquire PXRD patterns of the two Sor-Tos crystal habits (Rigaku, Tokyo, Japan). A rotating-anode Cu-target X-ray (λ = 1.5406 Å) worked at 40 kV, 250 mA, in the scanning range of 3.0 to 40.0° and scanning speed of 5°/min with an increasing step size of 0.02° and count time of 0.5~2 s. The calculated PXRD pattern of Sor-Tos was obtained from its SC-XRD data (CCDC No. 813503) [27] using Mercury 3.8 [29].

#### 3.3.4. X-ray Photoelectron Spectroscopy (XPS)

The surface chemistry of ST-A and ST-B was determined by XPS using a KRATOS AXIS ULTRA (DLD) spectrometer (Shimadzu, Japan). The binding energy was scanned in a range of 0–800 eV for regions of S 2p, Cl 2p, Cl 2s, C 1s, N 1s, O 1s, and F 1s, and their average binding energy was 165.2, 198.1, 270.9, 282.2, 397.4, 529.1, and 685.6 eV, respectively. Surface atomic concentration was calculated from the corresponding peak area.

#### 3.3.5. Molecular Modeling

The SYBYL-X 2.0 with the MOLCAD program was used to generate and visualize the lipophilic potential (LP) of Sor-Tos [30]. The computation was adjusted for small molecule simulation, and the Crippen table was set according to the study by Ghose et al. [31]. The surface of each group or atom exhibited the color ramp ranging from blue to red, which represented higher and lower LP, respectively. Visualization of molecular arrangement on the (300) crystal facet was analyzed using Mercury 3.8 [32].

#### 3.3.6. Face Indexation

The face indexation of a representative crystal of Sor-Tos was determined using a Bruker Smart Apex II-CCD diffractometer with Mo K_α_ radiation (λ = 0.71073 Å). Miller’s indices of various facets were recognized by T-tool, the face-indexing plug-in of Apex II [33].

### 3.4. Dissolution Rate Measurement

The dissolution rates of ST-A and ST-B in distilled water and gastric juice pH 1.2 acid solution (in the presence of 0.2% SLS) at 37 °C were measured using a Thermo Scientific Evolution 300 UV-Vis spectrometer (Thermo Scientific, Waltham, MA, USA). For dissolution rate measurement, an excess quantity of drug was poured into 200 mL of water or gastric juice pH 1.2 acid solution, which was pre-heated to 37 °C and rotated at 150 rpm. The mixture was stirred for 120 min, and at specific time intervals, 2 mL of the sample was withdrawn and replaced with 2 mL of a fresh medium to maintain a total volume. The sample was then filtered through Whatman’s 0.45 μm syringe filter. The absorbance at λ_max_ of the obtained solution was determined. The concentrations of ST-A and ST-B were achieved by calculations based on the standard curves of Sor-Tos. Each concentration was measured three times in parallel and the average value was obtained.

### 3.5. In Vivo Pharmacokinetics

A total of 36 male BALB/c athymic nude mice were randomly divided into two groups (18 for each group) for ST-A and ST-B. The mice were fasted for 12 h, then orally administered with Sor-Tos at a dose 27.5 mg kg^−1^. Blood was collected from the orbital sinus with a heparinized syringe at 0.5, 1, 2, 3, 4, and 8 h post drug administration. Blood was first centrifuged at 8000 rpm for 5 min, and 0.16 mL of the plasma was extracted. Then trifluoroacetic acid was used to precipitate protein, and NaOH was used to neutralize the solution. The mixture was diluted using ACN:H_2_O (7:3). After 10 min of precipitation, supernatant fluids were collected by centrifugation at 8000 rpm for 5 min, filtered with a syringe through a 0.45 μm hydrophilic membrane filter, and analyzed by the HPLC method. The experimental details are described in Supplementary Materials.

Interventional studies involving mice were performed in accordance with the China Animal Protection Law and were approved by the Zhejiang University Institutional Animal Care and Use Committee, China (approval ID ZJU20190018).

## 4. Conclusions

To improve the dissolution rate of sorafenib tosylate crystals without altering its structure, two crystal habits were prepared. The plate- and needle-shaped crystal habits of Sor-Tos were recrystallized from acetone and n-butanol. The two crystal habits showed identical structures but different surface anisotropy and abundance of exposed elements. The crystal surface of ST-B was more polar than that of ST-A, resulting in a faster dissolution rate and better in vivo pharmacokinetic behaviors of ST-B. Our study suggests that modifying the crystal habit is a good strategy to enhance the dissolution rate and pharmacokinetic performance of insoluble drugs, especially BCS class II drugs.

## Figures and Tables

**Table 1:** Percentage elemental composition on the surface of ST-A and ST-B.

Crystal Habit	Elemental Composition (%)						(O + N + S)/(C + F + Cl) (%)
	C	F	Cl	S	O	N	
ST-A	42.17	16.66	7.82	3.38	20.41	9.56	50.0
ST-B	41.18	17.42	7.33	3.75	20.13	10.20	51.7

**Table 2:** Pharmacokinetic parameters of Sor-Tos after a single dose at 27.5 mg kg^−1^ via oral administration.

Value	AUC (μg h mL^−1^)	*C*_max_ (μg mL^−1^)	*T*_max_ (h)
**ST-A**	9.33	1.85	3
**ST-B**	11.93	2.41	2

## Data Availability

The data presented in this study are available in the article.

## Supplementary Materials

The following are available online at Supporting Information.

## Sample Availability

Samples of the compounds sorafenib-tosylate are available from the authors.

## References

[1] Tran P., Pyo Y.-C., Kim D.-H., Lee S.-E., Kim J.-K., Park J.-S. (2019). Overview of the Manufacturing Methods of Solid Dispersion Technology for Improving the Solubility of Poorly Water-Soluble Drugs and Application to Anticancer Drugs. Pharmaceutics.

[2] Sathisaran I., Dalvi S.V. (2018). Engineering Cocrystals of Poorly Water-Soluble Drugs to Enhance Dissolution in Aqueous Medium. Pharmaceutics.

[3] Poovi G., Damodharan N. (2018). Lipid nanoparticles: A challenging approach for oral delivery of BCS Class-II drugs. Futur. J. Pharm. Sci..

[4] Pauli G., Tang W.-L., Li S.-D. (2019). Development and Characterization of the Solvent-Assisted Active Loading Technology (SALT) for Liposomal Loading of Poorly Water-Soluble Compounds. Pharmaceutics.

[5] Chen F., Fang Y., Zhao R., Le J., Zhang B., Huang R., Chen Z., Shao J. (2019). Evolution in medicinal chemistry of sorafenib derivatives for hepatocellular carcinoma. Eur. J. Med. Chem..

[6] Iyer R., Fetterly G., Lugade A., Thanavala Y. (2010). Sorafenib: A clinical and pharmacologic review. Expert Opin. Pharmacother..

[7] Porta C., Paglino C., Imarisio I., Ferraris E. (2009). Sorafenib tosylate in advanced kidney cancer: Past, present and future. Anti-Cancer Drugs.

[8] Gadaleta-Caldarola G., Infusino S., Divella R., Mazzocca A., Rose F.D., Filippelli G., Brandi M., Ferraro E., Abbate I. (2015). Sorafenib: 10 years after the first pivotal trial. Future Oncol..

[9] Wang X.-Q., Fan J.-M., Liu Y.-O., Zhao B., Jia Z.-R., Zhang Q. (2011). Bioavailability and pharmacokinetics of sorafenib suspension, nanoparticles and nanomatrix for oral administration to rat. Int. J. Pharm..

[10] Jiang S., Qin Y., Wu S., Xu S., Li K., Yang P., Zhao K., Lin L., Gong J. (2016). Solubility Correlation and Thermodynamic Analysis of Sorafenib Free Base and Sorafenib Tosylate in Monosolvents and Binary Solvent Mixtures. J. Chem. Eng. Data.

[11] Savjani K.T., Gajjar A.K., Savjani J.K. (2013). ChemInform Abstract: Drug Solubility: Importance and Enhancement Techniques. ISRN Pharm..

[12] Yang G., Kubota N., Sha Z., Louhi-Kultanen A.M., Wang J. (2006). Crystal Shape Control by Manipulating Supersaturation in Batch Cooling Crystallization. Cryst. Growth Des..

[13] Serrano D.R., O’Connell P., Paluch K.J., Walsh D., Healy A.M. (2016). Cocrystal habit engineering to improve drug dissolution and alter derived powder properties. J. Pharm. Pharmacol..

[14] Miletic T., Kyriakos K., Graovac A., Ibric S. (2013). Spray-dried voriconazole–cyclodextrin complexes: Solubility, dissolution rate and chemical stability. Carbohydr. Polym..

[15] Rasenack N., Müller B.W. (2004). Micron-Size Drug Particles: Common and Novel Micronization Techniques. Pharm. Dev. Technol..

[16] Sun C., Grant D.J.W. (2001). Influence of crystal shape on the tableting performance of L-lysine monohydrochloride dihydrate. J. Pharm. Sci..

[17] Banga S., Chawla G., Varandani D., Mehta B.R., Bansal A.K. (2010). Modification of the crystal habit of celecoxib for improved processability. J. Pharm. Pharmacol..

[18] Tari T., Szabó-Révész P., Aigner Z. (2019). Comparative Study of Different Crystallization Methods in the Case of Cilostazol Crystal Habit Optimization. Crystals.

[19] Laad P., Shete G., Modi S.R., Bansal A.K. (2013). Differential surface properties of commercial crystalline telmisartan samples. Eur. J. Pharm. Sci..

[20] Modi S.R., Dantuluri A.K.R., Puri V., Pawar Y.B., Nandekar P., Sangamwar A.T., Perumalla S.R., Sun C.C., Bansal A.K. (2013). Impact of Crystal Habit on Biopharmaceutical Performance of Celecoxib. Cryst. Growth Des..

[21] Ren Y., Shen J., Yu K., Phan C.U., Chen G., Liu J., Hu X., Feng J. (2019). Impact of Crystal Habit on Solubility of Ticagrelor. Crystals.

[22] Nokhodchi A., Bolourtchian N., Dinarvand R. (2005). Dissolution and mechanical behaviors of recrystallized carbamazepine from alcohol solution in the presence of additives. J. Cryst. Growth.

[23] Kumar D., Thipparaboina R., Sreedhar B., Shastri N.R. (2015). The role of surface chemistry in crystal morphology and its associated properties. CrystEngComm.

[24] Virupaxappa B.S., Shivaprasad K.H., Kulkani R.M. (2012). Latha, Research Journal of Pharmaceutical, Biological and Chemical Sciences. Hydroll. Process..

[25] Katritzky A.R., Chen K., Wang Y., Karelson M., Lucic B., Trinajstic N., Suzuki T., Schuurmann G. (2000). Prediction of liquid viscosity for organic compounds by a quantitative structure±property relationship. J. Phys. Org. Chem..

[26] Yang P., Qin C., Du S., Jia L., Qin Y., Gong J., Wu S. (2019). Crystal Structure, Stability and Desolvation of the Solvates of Sorafenib Tosylate. Crystals.

[27] Ravikumar K., Sridhar B., Rao A.K.S.B., Reddy M.P. (2011). Sorafenib and its tosylate salt: A multikinase inhibitor for treating cancer. Acta Crystallogr. Sect. C Cryst. Struct. Commun..

[28] Kiang Y.-H., Yang C.-Y., Staples R.J., Jona J. (2009). Crystal structure, crystal morphology, and surface properties of an investigational drug. Int. J. Pharm..

[29] Ruiz-Esparza G.U., Wu S., Segura-Ibarra V., Cara F.E., Evans K.W., Milosevic M., Ziemys A., Kojic M., Meric-Bernstam F., Ferrari M. (2014). Polymer Nanoparticles Encased in a Cyclodextrin Complex Shell for Potential Site- and Sequence-Specific Drug Release. Adv. Funct. Mater..

[30] (2011). Sybyl-X 2.1, Certara L.P. Louis, MO, USA. https://www.certara.com/pressrelease/certara-enhances-sybyl-x-drug-design-and-discovery-software-suite/.

[31] Ghose R., Nijhof V., Brouwer J., Matsubara Y., Kaida Y., Takahashi T. (1998). Shallow to very shallow, high-resolution reflection seismic using a portable vibrator system. Geophysics.

[32] Xia D., Cui F., Piao H., Cun D., Piao H., Jiang Y., Ouyang M., Quan P. (2010). Effect of Crystal Size on the In Vitro Dissolution and Oral Absorption of Nitrendipine in Rats. Pharm. Res..

[33] (2016). APEX3, SADABS and SAINT Bruker AXS Inc.: Madison, WI, USA. https://www.bruker.com/en/products-and-solutions/diffractometers-and-scattering-systems/single-crystal-x-ray-diffractometers/sc-xrd-software/apex.html.

