# Supplementary material for: Impact of Crystal Habit on the Dissolution Rate and In Vivo Pharmacokinetics of Sorafenib Tosylate
Source: Molecules. 2021 Jun 7;26(11):3469. doi: 10.3390/molecules26113469 (PMC8201088; doi:10.3390/molecules26113469)
Supplement: Supplementary file 1 [file molecules-26-03469-s001.zip › molecules-1209504-SM-revised-done.pdf]

molecules

Supporting information

Impact of Crystal Habit on the Dissolution Rate and In Vivo Pharmacokinetics of Sorafenib Tosylate

Chi Uyen Phan [1,*], Jie Shen [2], Kaxi Yu [2], Jianming Mao [2] and Guping Tang [2,*]

1 Faculty of Chemical Technology—Environment, The University of Danang—University of Technology and Education, Danang 550000, Vietnam
2 Department of Chemistry, Zhejiang University, Hangzhou 310028, China; shenjie1003@zju.edu.cn (J.S.); yukaxi@zju.edu.cn (K.Y.); 3150102050@zju.edu.cn (J.M.)
* Correspondence: pcuyen@ute.udn.vn (C.P.); tangguping@zju.edu.cn (G.T.); Tel.: +84 0962119542 (C.P.).

Citation: Phan, C. U.; Shen, J.; Yu, K.; Mao, J.; Tang, G. Impact of Crystal Habit on the Dissolution Rate and In Vivo Pharmacokinetics of Sorafenib Tosylate. *Molecules* **2021**, 263469. https://doi.org/10.3390/molecules26113469

Academic Editors: Guy Van den Mooter and Korbinian Löbmann

Received: 20 April 2021
Accepted: 5 June 2021
Published: date

Publisher's Note: MDPI stays neutral with regard to jurisdictional claims in published maps and institutional affiliations.

Copyright: © 2021 by the authors. Submitted for possible open access publication under the terms and conditions of the Creative Commons Attribution (CC BY) license (http://creativecommons.org/licenses/by/4.0/).

Results and discussion

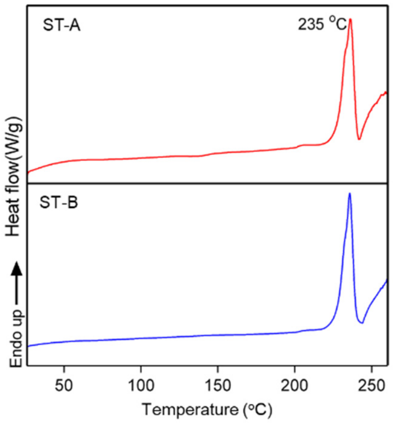

Figure S1. Overlay of DSC curves of ST-A and ST-B.

Table S1. The PXRD relative intensity (I/I0, %) of Sor-Tos crystal habits and calculated from single-crystal diffraction results.

d (Å)	2 theta (°)	(hkl)	ST-B	ST-A
19.99	4.4	(100)	30.3	48.5
8.02	11.0	(10-2)	7.4	9.6
6.69	13.2	(300)	100	100
5.99	14.7	(11-2)	15.3	23.5
5.31	16.6	(202)	20.1	41.9
4.96	17.8	(40-2)	27.4	43.0
4.34	20.4	(12-1)	27.9	41.6
4.26	20.8	(31-3)	28.4	37.9
4.12	21.5	(50-2)	37.5	61.6
3.89	22.9	(221)	33.9	54.2

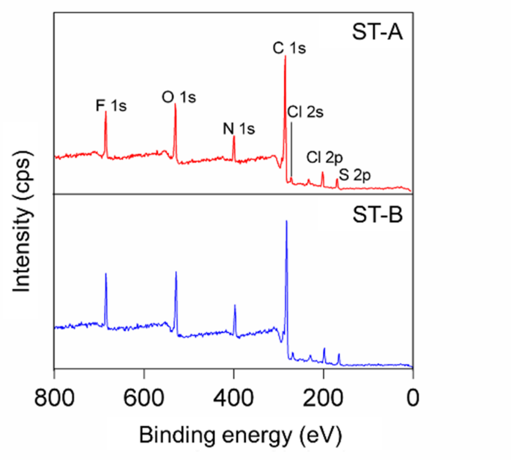

Figure S2. Overlay of XPS spectra of ST-A and ST-B.

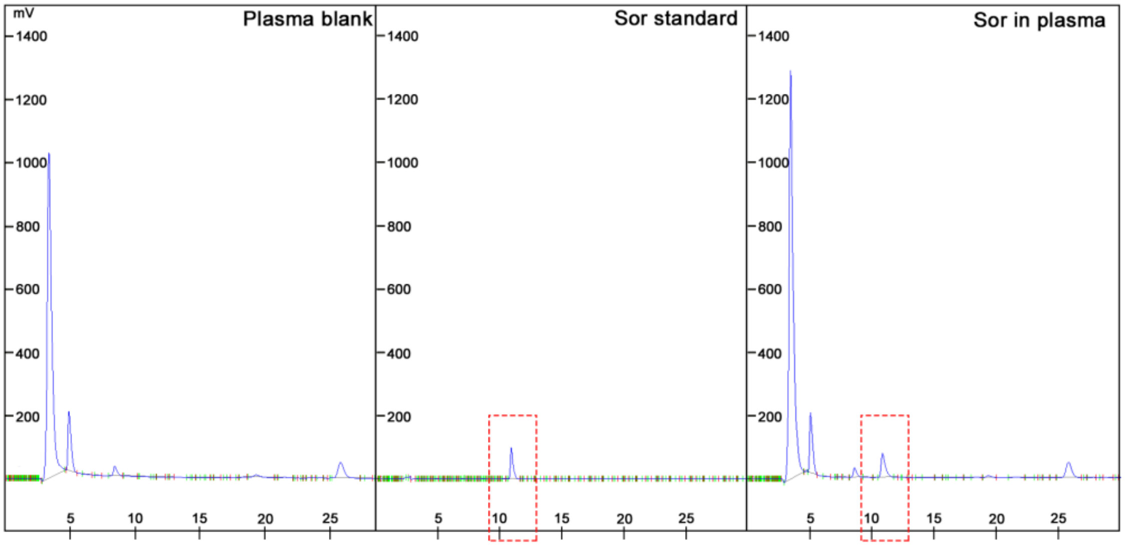

Figure S3. The HPLC chromatogram of plasma blank, Sor standard and Sor in plasma.

The HPLC chromatogram of plasma blank, Sor standard and Sor in plasma were reported. The results showed that, plasma blank HPLC chromatogram exhibited protein peaks at retention time of 3.34 min, 4.85 min, 8.33 min and 25.80 min. HPLC chromatogram of Sor standard while displayed one peak at retention time of 10.81 min, and Sor in plasma HPLC chromatogram obviously presented peaks corresponding to the retention time of Sor standard and plasma blank.

The standard curve of five concentrations and absorbance of Sor were obtained (Figure S4), it was linear in a concentration range of 0.2 – 1.2 μg mL^{-1}, in which y = 2.956e+006*x + 270507, R^2 = 0.9986. In the case of Sor in plasma, its standard curve was also linear with the correlation coefficients of all linear-regression lines was 0.9918, and y = 2.788e+006*x + 60129. The results showed that a small amount of Sor was absorbed by protein in plasma. All in vivo pharmacokinetic was calculated based on the standard curve of Sor in plasma.

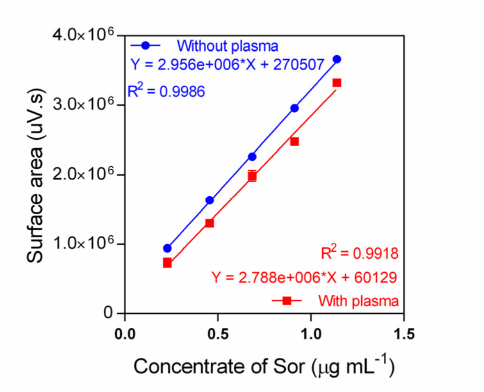

Figure S4. Standard curves of Sor in ACN:water (7:3) mixture with and without plasma.

Materials and Methods

HPLC assay

HPLC method was used for determining the concentration of Sor in the in vivo pharmacokenitic experiment. The analytical column was a Agilent ZORBAX-SB-C18 column (4.6 mm × 150 mm, 5 μm). The mobile phase was ACN: H_2O (7:3 v/v), the flow rate of mobile phase was 0.8 mL min^{-1} and the UV detector was at 265 nm.

Sor in plasma samples were prepared in plasma from BALB/c mouse, which were not undergoing treatment with Sor. Plasma was separated from 2 mL blood by centrifugation at 8000 rpm for 5 min. Then 1 mL stock solution of Sor was added into 0.75 mL plasma. After mixing for 5 min, 20 μL CF_3COOH was added to deproteinization. The mixture was neutralized by NaOH 1 mol L^{-1} and centrifuged at 8000 rpm for 5 min to separate precipitated protein. The solution was then diluted into a 10 mL volumetric flask using ACN:H_2O (7:3). Then a set of calibration standards is prepared by pipetting 0.1 mL, 0.2 mL, 0.3 mL, 0.4 mL and 0.5 mL of the solution into separate 10 mL volumetric flasks and diluting with ACN:H_2O (7:3) solution. The standard curve of Sor in plasma was prepared by plotting the peak area ratio to Sor at these 5 points, and linearity was assessed by the linear-square method. Blank plasma sample was prepared through these same steps without added stock solution of Sor. The standard curve of Sor in solvent was prepared in ACN:H_2O (7:3) solution without adding into plasma. The corresponding standard curve of the concentration and peak area of Sor was also obtained, and linearity was assessed by the linear-square method. Each concentration was measured three times in parallel and the average value was obtained.
